# A dilemma of a case of Zenker diverticulum; leak or Acinetobacter baumannii?! A case report

**DOI:** 10.1016/j.ijscr.2020.05.004

**Published:** 2020-05-29

**Authors:** Abdulwahid M. Salih, Twana M. Ameen, Safeen O. Mahmood, Fahmi H. Kakamad, Bahman L. Fathulla, Tomas M. Mikael, Imad J. Habibullah, Shvan H. Mohammed, Rawezh Q. Salih, Suhaib H. Kakamad

**Affiliations:** a Faculty of Medical Sciences, School of Medicine, University of Sulaimani, François Mitterrand Street, Sulaimani, Kurdistan, Iraq; b Smart Health Tower, Sulaimani, Madam Mitterrand Street, Kurdistan, Iraq; c Shar Teaching Hospital, Malik Mahmud Ring Road, Sulaimani, Kurdistan, Iraq; d Kscien Organization for Scientific Research, Hamdi Street, Sulaimani, Kurdistan, Iraq; e Sulaymaniyah Teaching Hospital, François Mitterrand Street, Kurdistan, Iraq

**Keywords:** Acinetobacter baumannii, Leak, Zenker’s diverticulum

## Abstract

**Introduction:**

Zenker’s diverticulum is an acquired mucosal pulsion of the upper esophagus. The aim of this paper is to report a case of Zenker’s diverticulum with a complicated postoperative course including infection by Acinetobacter baumannii causing diagnostic as well as management dilemma.

**Case report:**

A 43-year-old male complained from dysphagia for three months. Barium swallow revealed Zenker’s diverticulum. Under general anesthesia, the small Zenker’s diverticulum was ligated and myotomy was done. He developed erythematous swelling at the site of the operation. The patient was taken to the operation theater. The esophagus was tested, no trace of leak was found. The patient developed cardiac arrest. He was admitted to the intensive care unit and remained there for twenty eight days. Bronchial wash culture showed Acinetobacter baumannii. The patient was on antibiotic, supportive treatment and enteral feeding through nasogastric tube. After recovery he was extubated and barium swallow was done which was normal and showed no signs of leak. Three months after the operation, the patient was completely normal.

**Discussion:**

This case had a positive culture for Acinetobacter baumannii. It is commonly accounted for nosocomial infections. It has been documented as a serious threat worldwide because of the emerging prevalence of multidrug resistant. However, an inadequate understanding of A. baumannii pathophysiology and ecosystem confines the progress of alternative therapeutic policies. In the current case, it was not clear either leak or Acinetobacter baumannii was the cause of deterioration.

**Conclusion:**

Both esophageal leak and infection with Acinetobacter baumannii are fulminant debilitating conditions that could be managed with conservative strategies.

## Highlights

• Zenker’s diverticulum is an acquired mucosal pulsion of the upper esophagus.
• Post operative leak and infection could lead to a dilemma to both surgeon and patient.
• We reported a case of Zenker’s diverticulum with a complicated postoperative course.

## 1 Introduction

Zenker’s diverticulum (ZD) is defined as an acquired mucosal pulsion through the Killian triangle, the triangle is bounded by the oblique fibers of the inferior pharyngeal constrictor and the horizontal fibers of the cricopharyngeal muscle. The condition is more common in middle and elderly age. The most prevalent presenting symptom is dysphagia followed by regurgitation, choking, halitosis, cough, aspiration, chest infection and esophageal obstruction [1]. Myotomy is the principle of therapy which can be performed with flexible or rigid endoscope or with open surgery. The latter has the advantage of feasibility of diverticulectomy. Infection is a relatively common postoperative complication [2].

Acinetobacter is a group of Gram‐negative proteobacteria with unusual metabolic diversity. Some of the species live alone in the environment, while many of them comprise a critical part of the human microbiome and in some situation they are opportunistic pathogens like Acinetobacter baumannii [3]. Acinetobacter baumannii is a challenging nosocomial infection causing a wide range of diseases starting from simple urinary tract infection to fatal and progressive soft tissue infection, especially among immunocompromised individuals [4].

The aim of this paper is to report a case of ZD with a complicated postoperative course including infection by Acinetobacter baumannii causing diagnostic as well as management dilemma. The report has been arranged in line with SCARE guidelines [5].

**Patient Information:** A 43-year-old male complained from dysphagia and halitosis for three months, which was gradual and progressive in nature for both liquid and solid foods. It was associated with regurgitation of indigested food, hiccup, halitosis, productive cough and drooling of saliva during sleep. It was not associated with fever, anorexia or weight loss. The patient had history of lower limb superficial varicosities, sacral vertebrae disc prolapse (S4-S5) and Graves’ disease which was treated surgically (total thyroidectomy three years before this presentation) since that time, he had been put on thyroxin tablet 100 microgram once daily. He was a cigarette smoker with 13 pack-year smoking history. He had no family history of the same condition.

**Clinical Findings:** Clinical examination was negative. There was no significant finding in respiratory, cardiovascular, elementary, skin and musculoskeletal systems.

**Fig. 1:**
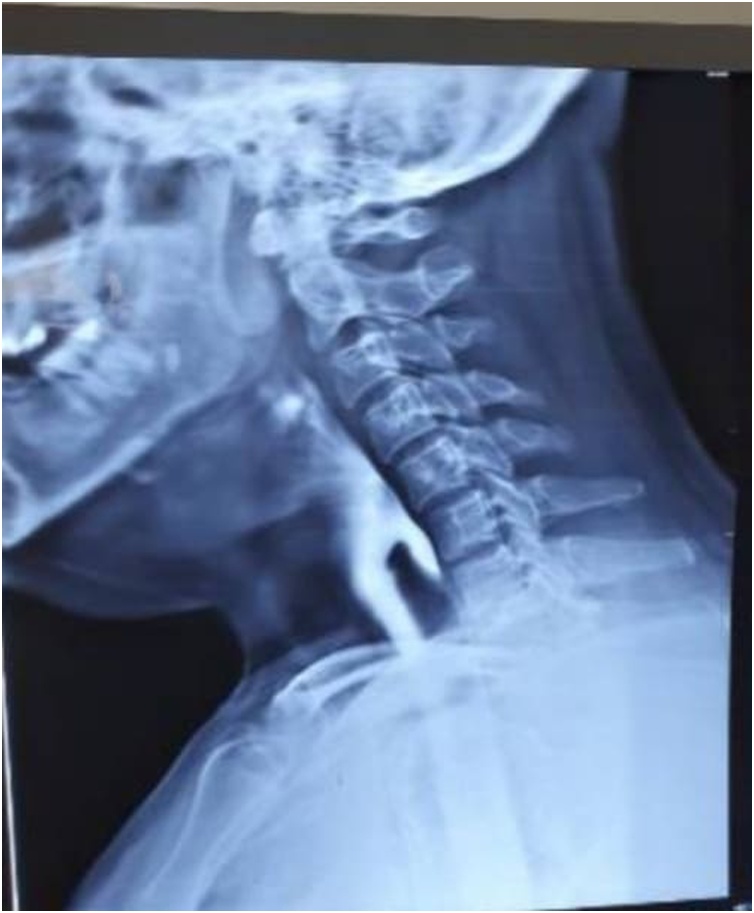
Barium swallow showing Zenker’s Diverticulum.

**Diagnostic Assessment:** Chest X-ray and complete blood counts were normal. Barium swallow and esophagogastrodudenoscopy (OGD) revealed findings consistent with ZD (Fig. 1). The patient was prepared for general anesthesia.

**Therapeutic Intervention:** Under general anesthesia, in supine position, through the old cervical incision (previous collar incision for thyroidectomy), strap muscles were separated, left sternocleidomastoid muscle retracted laterally. A small (2 cm) ZD was found and ligated in flush with the esophagus and cricopharygeal myotomy was performed. The patient recovered in a good condition. Four hours after operation he could take liquid food without any difficulty.

**Follow up:** In the first day of operation, the patient developed nausea, fever, rigor and sever constant pain which did not respond to oral analgesia. Then he developed erythematous swelling at site of the operation which was associated with bloody discharge from the surgical stiches and hotness.

**Fig. 2:**
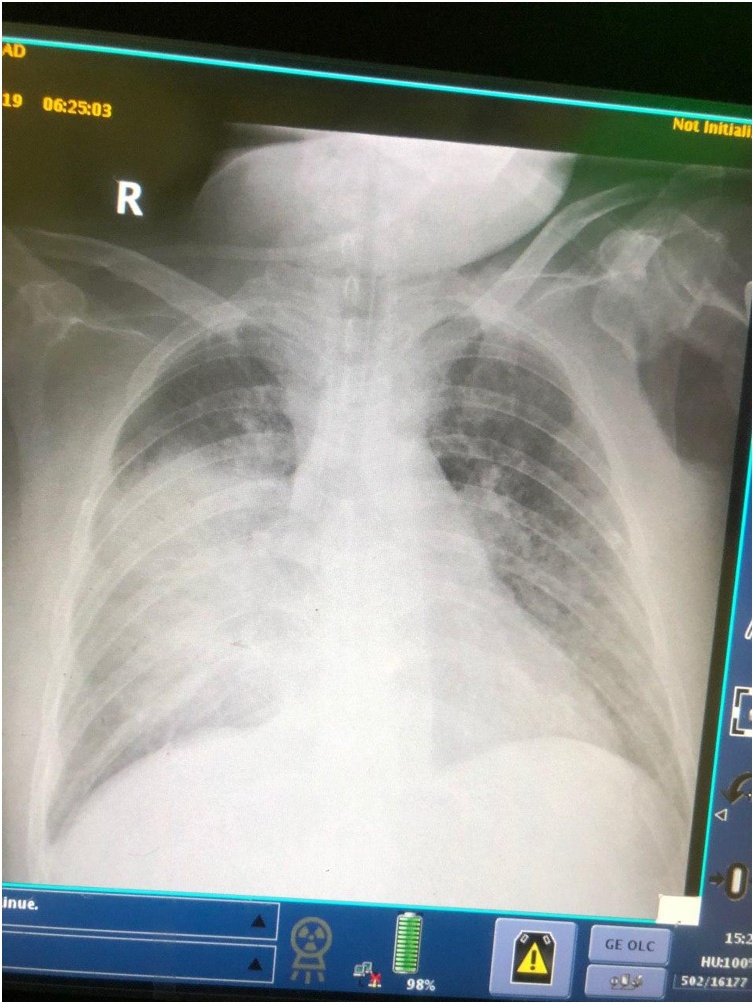
Chest x.ray 15 days after operation showing right lung collapse.

On examination the patient had high temperature, tachycardia and hypotension. Then he deteriorated more and his consciousness level altered. There was progressive swelling at the site of the operation. The patient was taken to the operation theater, minimal amount of hematoma was found. The esophagus was tested using air and saline for perforation, no bridge was found, later re-assessed by diluted methylene blue, again no trace of leak was found. The patients was recovered and extubated, few minutes later, he deteriorated and developed cardiac arrest, he was revived by cardiopulmonary resuscitation (CPR). The patient was admitted to intensive care unit (ICU) and remained there for twenty eight days, during that period he developed right side lung collapse (Fig. 2). In the 7th postoperative day, a tracheostomy was created. Computed tomography scan showed soft tissue swelling in the left anterior chest wall, there was no sign of mediastinitis. He was managed by six sessions of bronchoscopy, lavage and wound care. He was put on nasogastric tube feeding. Bronchial wash culture showed Acinetobacter baumannii. The patient was put on antibiotic, supportive treatment and enteral feeding through nasogastric tube. After recovery he was extubated and barium swallow showed no leak. Then tracheostomy tube inserted and put on supportive measures including daily lavage, suctioning and wound care.

When the patient’s condition become better, he discharged from ICU and admitted to surgical ward for seven days. After two days, tracheostomy tube removed.

The patient could take orally without any difficulty but he complained of dyspnea on exertion, productive cough, mild fever and easy fatigability. He was managed by antibiotics, expectorant and physiotherapy. Three month after the operation, the patient was completely normal.

## 2 Discussion

Zenker Diverticulum has been attributed to the old age men while the current case was a middle age male patient [2]. The presentation and diagnostic work up for this case was the same as reported in the literature [1].

The options for treatment embrace transcervical cricopharyngeal myotomy, minimally invasive endoscopic myotomy, and transoral rigid endoscopic stapling. Although endoscopic procedures have become more and more performed, open technique is still the standard for management of ZD [6]. The current case underwent open procedure with myotomy and ligation of the pouch. Efficacious surgical management can be accomplished in 80%–100%; however, morbidity (30%) and mortality (3%), have been reported [7]. Several cases undergoing esophageal operation hurt from postoperative complications, and these problems are frequently life threatening. This is the case when the esophagus has been opened and repaired or anastomosed, because the esophageal anastomosis is more liable for leak than any other parts of gastrointestinal tract [8]. Postoperative leaks have various severities ranging from asymptomatic that is only apparent on imaging, to fulminant presentation with sepsis and multi-organ failure. The cause of esophageal leak is multifactorial mainly due to ischemia and conduit failure. In spite of many advances in surgical techniques and technologies, esophageal anastomotic leak remains a significant and frequent source of postoperative morbidity and mortality [8]. The strange finding in this case was that, there was no sign of leak, neither radiologically nor by native eyes intraoperatively in spite of that the patient deteriorated after an oral intake.

This case had positive culture for Acinetobacter baumannii, it is a gram negative opportunistic pathogen that has no specific niche. It is commonly accounted for nosocomial infections, especially hospital acquired respiratory infection, septicemia, urinary tract infections (UTI) and soft tissue infections [9]. Rarely, community acquired infections have been reported caused by A. baumannii. Furthermore, it is documented as a serious threat worldwide because of the emerging prevalence of multidrug resistant (MDR). However, an inadequate understanding of A. baumannii pathophysiology and ecosystem confines the progress of alternative therapeutic policies [9]. In the current case, it was not known either leak or Acinetobacter baumannii was the cause of deterioration.

In conclusion, both esophageal leak and infection with Acinetobacter baumannii are fulminant debilitating conditions that could be managed with conservative strategies.

## Author contribution

Abdulwahid M. Salih: Surgeon performing the operation, final approval of the manuscript and follow up.

Twana Muhamad Amin: anaesthesiologist following up the patient, final approval of the manuscript.

Safeen Othman Mahmood: clinical microbiologist; diagnosing and managing the patient, final approval of the manuscript.

Fahmi H. Kakamad, Bahman L. Fathulla, Tomas M. Mikael, Rawezh Q. Salih: Writing the manuscript, final approval of the manuscript and follow up.

Imad J. Habibullah, Shvan H.Mohammed, Suhaib H.Kakamad: literature review, final approval of the manuscript.

## Informed consent

A written informed consent was taken from the patient and patient’s family for publication of this report.

## Ethical approval

Approval is not necessary for case report in our locality.

## Consent

Consent has been taken from the patient and the family of the patient.

## Registration of research studies

Not applicable.

## Guarantor

Fahmi Hussein Kakamad.

## Funding

No source to be stated.

## Provenance and peer review

Not commissioned, externally peer-reviewed.

## Declaration of Competing Interest

None to be declared.
